# Evaluating the geographic distribution of rural PFAS contamination and potential dietary sources of exposure

**DOI:** 10.1038/s44454-026-00055-z

**Published:** 2026-09-02

**Authors:** Haeseung Yi, Crystal So, Tammy A. Flores, Haley L. Motola, Jill Hubert-Simon, Stella Yi, Vittorio Albergamo, Lorna E. Thorpe, Leonardo Trasande, David C. Lee

**Affiliations:** 1 Department of Emergency Medicine, NYU Langone Health, New York, NY USA; 2 Sullivan County Public Health Services, Liberty, NY USA; 3 Department of Population Health, NYU Langone Health, New York, NY USA; 4 Department of Pediatrics, NYU Langone Health, New York, NY USA

**Keywords:** Environmental sciences, Health care, Risk factors

## Abstract

Aside from specific occupational exposures, ingestion is believed to be the primary route of exposure to PFAS, a class of forever chemicals known for their ability to repel oil and water, reduce friction, and resist high temperatures. The goal was to explore the geographic distribution of PFAS water contamination versus human exposure and identify dietary factors that may explain high PFAS levels among rural residents. We performed PFAS analyses among 160 rural participants, who provided blood samples and survey responses in our chemical exposure cross-sectional study. We mapped the geographic distribution of PFAS contamination based on water testing versus human samples results. We also used health surveys and food frequency questionnaire data to identify factors associated with high PFAS levels. The geographic mismatch between the distribution of PFAS water contamination and human exposure highlights the need to assess other potential sources, which identified potential dietary sources of these chemical hazards.

## Introduction

Concerns about PFAS contamination started in the late 1990s, and its impact on human health has been a growing area of research ever since^1^. Despite these concerns, production of products containing PFAS have been difficult to curb due to their unique, highly functional properties—they repel oil and water, reduce friction, and are heat resistant^2^. Therefore, they have been used in a wide variety of common products, including non-stick cookware, food packaging, stain and water-resistant clothing and fabrics, firefighting foams, paints, cleaning products, and personal care products (e.g., shampoos, dental floss, and cosmetics)^3^. While chemical manufacturers have phased out the use of some PFAS chemicals, concerns about modern iterations of legacy PFAS, and their potential health effects, have emerged^4^.

PFAS (per/polyfluoroalkyl substances) are often called forever chemicals because of their resistance to degradation due to a carbon-fluorine bond^1,5,6^. As a result, PFAS persists in the environment and accumulates in humans and other lifeforms. They are also ubiquitous, making it difficult to detect the key sources that cause exposures. While the health effects of PFAS chemicals continue to be studied, there is sufficient evidence demonstrating their negative impact on human health^3^. In 2021, a committee convened by the National Academies of Sciences, Engineering, and Medicine, found literature associating PFAS chemicals with decreased immune response, high blood lipid levels, decreased infant and fetal growth, increased risk of kidney cancer, and other health problems^1^.

Given that ingestion is considered the primary route of PFAS exposure, surveillance of contamination has largely been focused on water source testing, conducted by state and local government agencies, as well as periodic total diet sampling conducted by the Food and Drug Administration^7^. Relatedly, there has been less emphasis on testing humans for PFAS exposure as a means to determine the relative contributions of various sources and their impact on human health^1^. Therefore, the goals of this study were twofold: (1) to explore the geographic distribution of PFAS water contamination versus human exposure in a rural county with known PFAS cleanup sites, and (2) to identify dietary sources of chemical hazards among rural residents by testing human samples for PFAS exposure.

## Results

### Study participants

Of the 160 rural residents whose blood samples were tested for PFAS, the median age was 63 years, with a wide age range from 25 to 93 years old. Study participants were 65% female and 86% non-Hispanic White, which is broadly comparable to Census estimates for Sullivan County in 2023 at 48% female and 67% non-Hispanic White (Table 1). There were no statistically significant differences between these demographic and socioeconomic attributes when compared among the tertiles for total PFAS exposure.

**Table 1:** Characteristics of the study population in Rural Sullivan County, New York

Characteristics	All participants	Upper tertile	Middle tertile	Lower tertile	Significance *p* value
Participants	160	54	53	53	
**Total PFAS** (ng/mL)					
Mean	9.1	14.8	8.1	4.3	
Median	8.0	14.0	7.9	4.7	
Range	1.5–33.1	10.9–33.1	5.9–10.9	1.5–5.9	
**Age**					
Mean	59	58	60	58	0.64
Median	63	66	63	59	
Range	25–93	25–93	31–85	28–84	
**Sex**					
Male	35%	55%	36%	16%	0.46
Female	65%	45%	64%	84%	
**Race/Ethnicity**					
NH White	86%	88%	85%	86%	0.09
NH Black	3%	2%	4%	2%	
Hispanic	9%	6%	11%	10%	
Other	2%	4%	0%	2%	
**Household composition**					
Single without children	43%	43%	43%	43%	0.37
Single with children	8%	14%	6%	4%	
Married without children	38%	37%	40%	37%	
Married with children	11%	6%	11%	16%	
**Household Income**					
Less than $25,000	20%	20%	21%	20%	0.36
$25,000 to $49,000	22%	23%	28%	16%	
$50,000 to $99,999	26%	24%	25%	29%	
More than $100,000	32%	33%	26%	35%	
**Highest level of education**					
Some High School	3%	4%	2%	2%	0.79
High School Grad or GED	18%	12%	19%	25%	
Some					
College	24%	33%	21%	18%	
College Degree or More	55%	51%	58%	55%	

### PFAS human exposure data

Of the panel of PFAS chemicals tested, the most commonly present were PFOS-2 (sum of branched and linear isomers), PFOA, and PFHxS, which were detected in all 160 study participants. These three chemicals also had the highest average concentrations at 5.5 ng/mL for PFOS-2, 1.5 ng/mL for PFOA, and 1.1 ng/mL for PFHxS. Three other chemicals in the PFAS panel identified in most study participants were PFNA (98% of participants), PFUnDA (74%), and PFDA (53%). All other PFAS chemicals were detected in less than 20% of the study population. The average total levels of PFAS summed across all chemicals was 9.1 ng/mL with a range of 1.5–33.1 ng/mL.

### Geography of PFAS contamination and exposure

**Fig. 1:**
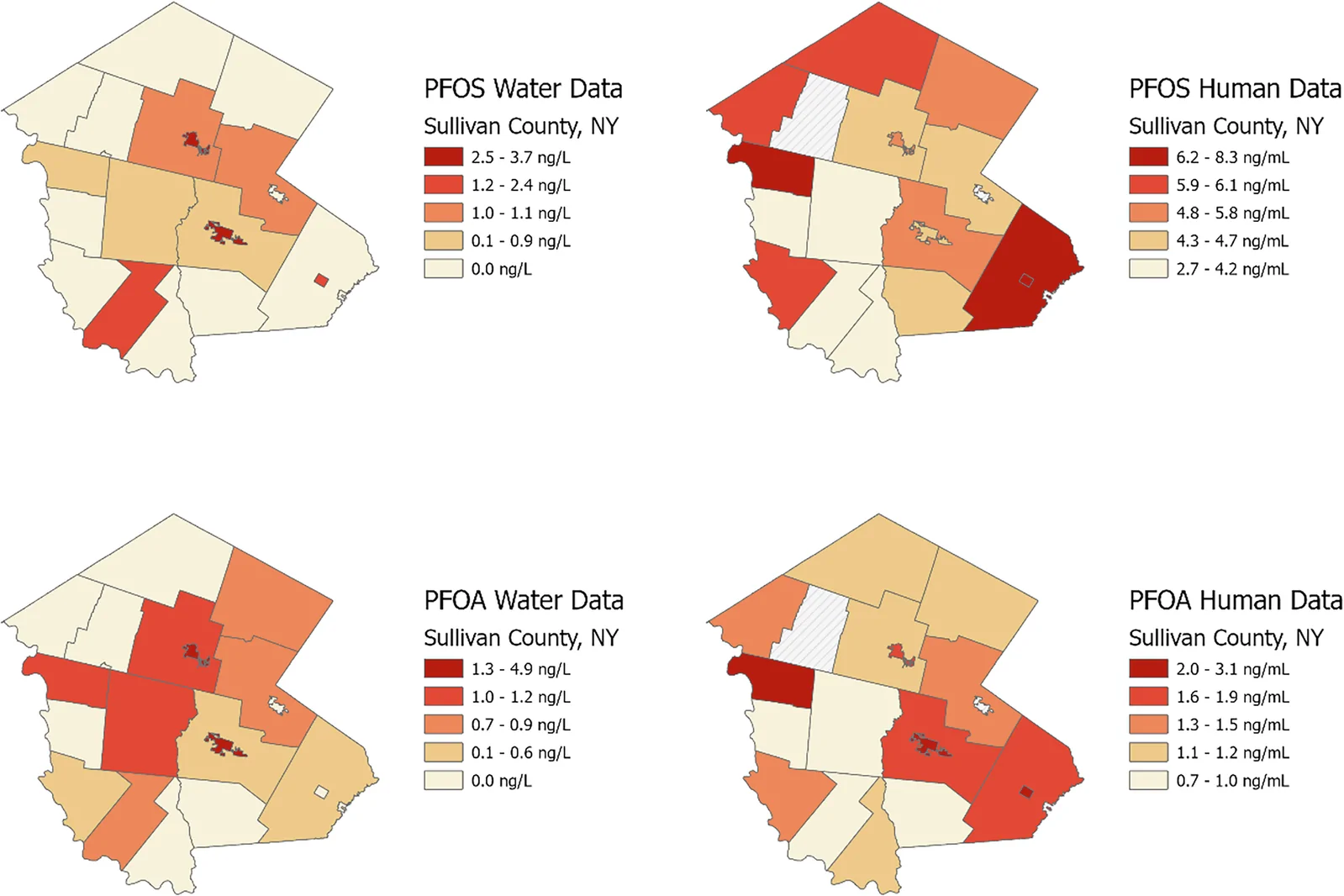
Geographic distribution of PFOS and PFOA contamination and exposure. Levels of PFOS and PFOA in water samples and human samples in Sullivan County as analyzed by Census subdivisions (e.g., towns and villages). Darker shaded areas represent areas with higher levels or exposure.

As depicted in Fig. 1, the geographic distribution of PFOS-2 and PFOA water source contamination was primarily concentrated in the central part of the county, which is more densely populated. However, the geographic distribution of PFOS-2 and PFOA exposure in human samples demonstrated higher average levels in areas different from those indicated by water testing results. Correlation was non-existent between PFOS-2 water contamination versus PFOS-2 human exposure by geographic area with a coefficient of –0.02 (*p* value = 0.93). Correlation was also poor between PFOA water contamination and PFOA human exposure by geographic area with a coefficient of 0.22 (*p* value = 0.40). Furthermore, we did not find higher levels of PFAS among residents who lived in geographic proximity to a known PFAS cleanup site at the local airport compared to residents who lived far from the site.

### Demographic, socioeconomic, and dietary factors associated with PFAS exposure

In our multivariable regression analysis using LASSO, we analyzed factors that predicted a high level of total PFAS. The strongest predictors were older age with the highest LASSO regression coefficient ( + 0.17) and male sex ( + 0.16). Notably, no other demographic or socioeconomic factors were predictive of high total PFAS levels. As for dietary factors, there were several statistically significant predictors of high total PFAS levels, which included intake of shellfish ( + 0.09), veal, lamb, goat, deer, or other game ( + 0.08), wine ( + 0.08), and cottage or ricotta cheese ( + 0.06). High intake of fresh fruit was one dietary factor associated with low total PFAS exposure (–0.05).

In addition, we also studied the six PFAS chemicals in our panel that were identifiable in most study participants. Notably, age was the only predictor that was statistically significant across all six of these PFAS chemicals. The second strongest predictor, male sex, was associated with higher levels of PFAS for only three specific chemicals: PFOS-2 ( + 0.16), PFOA ( + 0.09), and PFDA ( + 0.09). Being a high school graduate predicted low levels of PFUnDA (–0.14). Aside from these factors, no other demographic or socioeconomic factor predicted high levels for any of the six specific PFAS chemicals analyzed individually.

We also noted that some dietary factors were predictive of certain PFAS chemicals but not others. For instance, intake of shellfish and intake of veal, lamb, deer meat or game was predictive of high levels of PFOS-2 and other PFAS but not PFOA. Conversely, intake of wine was predictive of high levels of PFOA and other PFAS chemicals, it was not predictive for PFOS-2.

We also noted that PFUnDA was particularly high among those with a high intake of several seafood categories, such as shellfish, tuna, salmon, mackerel, sea bass, trout, sardines, herring, and other fish. There was also a strong association of high avocado intake with high levels of PFUnDA and PFDA. Other statistically significant predictors of both high and low total PFAS and specific PFAS are listed in Table 2. Some dietary factors had lower strength of association based on the magnitude of their LASSO coefficients or were only influential for one particular PFAS.

**Table 2:** Regression results identifying demographic, socioeconomic, and dietary factors associated with high PFAS levels

Associated Factors	Total	PFOS-2	PFOA	PFHxS	PFNA	PFUnDA	PFDA
*Mean Levels and Proportion with Detectable Levels*							
Limit of Detection (LOD)	N/A	0.010	0.026	0.014	0.015	0.012	0.016
Mean Levels (ng/mL)	9.1	5.5	1.5	1.1	0.5	0.1	0.1
Max Level (ng/mL)	33.1	22.9	7.4	10.8	3.9	0.6	0.7
Proportion Positive	**100%**	**100%**	**100%**	**100%**	**98%**	**74%**	**53%**
*Influential for Total PFAS*							
Older Age	**+0.17**	**+0.20**	**+0.10**	**+0.19**	**+0.22**	**+0.14**	**+0.15**
Male Sex	**+0.16**	**+0.16**	+0.09				+0.09
Shellfish	+0.09	**+0.13**			**+0.16**	**+0.12**	**+0.30**
Veal, Lamb, Goat, Deer, Other Game	+0.08	**+0.15**			**+0.10**		**+0.27**
Wine	+0.08		**+0.11**		**+0.21**	**+0.19**	**+0.12**
Cottage Cheese, Ricotta Cheese	+0.06	+0.06		+0.08			
Other Fresh Fruit	-0.05	**-0.11**			-0.06		
*Influential for More than One PFAS*							
Plain Pasta, Pasta Salad, Sopa Seca		-0.09			**-0.16**		**-0.17**
Sports Drinks					**-0.10**	-0.05	
Tuna or Tuna Salad					+0.08	**+0.23**	
Avocado, Guacamole						**+0.21**	**+0.23**
Pancakes, Waffles, French Toast,Crepes						**-0.10**	**-0.19**
Pumpkin Pie, Sweet Potato Pie						-0.08	**-0.11**
Soft Drinks, Soda						-0.05	**-0.11**
Gravy, Rich Sauces						-0.08	-0.09
*Influential for Only One PFAS*							
Milk on Cereal		+0.05					
Ice Cream		-0.05					
French Fries, Home Fries, Hash Browns, Tater Tots					-0.05		
Salmon, Mackerel, Sea Bass, Trout, Sardines, Herring						**+0.21**	
Meal Replacement Drinks						**-0.19**	
Other Fish						**+0.15**	
Strawberries or Other Seasonal Berries						**+0.14**	
High School Graduate or GED						**-0.14**	
Donuts						**-0.12**	
Green Beans, String Beans, Green Peas						**-0.10**	
Other Breads						-0.08	
Water						-0.08	
Vegetable Soup						-0.05	
Tofu or Tempeh							**-0.13**
Orange or Grapefruit Juice							**-0.11**
Eggs							**+0.10**
Kool-Aid, Lemonade							**-0.10**

Bold values are for a coefficient with an absolute value of 0.10 or higher.

## Discussion

Based on available PFAS water contamination data and human samples analyzed from our rural study of chemical exposures, we did not find any geographic overlap between areas with high PFAS water contamination and areas with high PFAS exposure among rural residents of Sullivan County, New York. We found that older age and male gender were the only major demographic factors associated with high PFAS exposure, whereas the socioeconomic factors tested did not have much meaningful influence. Our findings are consistent with another study that characterized PFAS exposure in a population residing near a point-source drinking water contamination in the Veneto Region of northern Italy and found that while water contamination was the primary determinant of serum PFAS levels, additional factors such as sex, years of residence, and raising own livestock significantly influenced internal dose, reinforcing the concept that PFAS body burden in contaminated communities is shaped by multiple exposure pathways beyond drinking water alone^8^. As for dietary predictors, we found that intake of shellfish, cottage or ricotta cheese, wine, and the category of veal, lamb, goat, deer or other game were associated with high PFAS among the rural residents studied.

Our findings do not suggest that the surveillance of water sources for PFAS is unimportant, especially given that some communities have been disproportionately affected by water contamination. Rather, our study suggests that dietary sources may contribute meaningfully to PFAS exposure in areas where drinking water contamination is not substantially high. In this study, the highest measured level of PFOA was 9.5 ng/L and the highest level of PFOS-2 was 8.9 ng/L, which are both above the EPA threshold of 4.0 ng/L for each PFAS chemical, but not as high as the levels measured in severely affected communities^9–11^. It is critical to note that while serum PFAS levels were measured at an individual level and compared to individual level dietary patterns, that drinking water PFAS levels were measured at an ecologic level and may not be indicative at all of actual PFAS exposure through water consumed by the individuals in our study. Therefore, the next stage of our study will be to specifically investigate the actual water sources ingested by study participants in our study.

Of dietary factors associated with high PFAS levels, we found shellfish and fish were the strongest predictors, especially for PFUnDA. The variation in predictors suggests that the risk of specific PFAS chemical exposures may have different dietary sources. Our findings support established literature, which demonstrates seafood to be the most concerning potential dietary source of PFAS exposure^7^.

Consumption of cottage or ricotta cheese was associated with high PFAS exposure, supporting several studies on potential PFAS contamination of dairy products^12–15^. In addition, we found an association between wine consumption and several PFAS chemicals. As of date, there are only a few studies suggesting this link: one study in Belgium found PFAS chemicals in wine samples, and another study in Spain demonstrated that regular consumption of wine had a positive association with high levels of PFOA, PFOS, PFNA, and PFDA^16,17^.

Notably, the category of veal, lamb, goat, deer meat, and game was a strong predictor of high PFAS exposure in our rural study population^18^. This particular finding may suggest a risk factor unique to the rural environment, where hunting for game is more common. In fact, the Department of Fisheries and Wildlife and Centers for Disease Control and Prevention in Maine has issued “Do Not Eat” advisories for game, such as deer and turkey, caught in certain areas known for PFAS contamination^19^. Our study suggest that this risk may extend beyond Maine and include other rural regions of the United States, where local game live in areas contaminated with PFAS.

When analyzed individually, a higher intake of other fresh fruit (e.g., grapes, plums, mango, and fruit salad) predicted low levels of total PFAS as well as PFOS-2 and PFNA. Other dietary factors associated with low PFAS exposure included plain pasta, pasta salad and sopa seca, and pancakes, waffles, French toast, and crepes. While it was not obvious how a higher intake of these foods might lead to lower PFAS exposure, it is possible that these categories of foods were proxies for dietary sources or other factors related to PFAS exposure. For instance, intake of meal replacement drinks was associated with lower levels of PFUnDA, which could mean that participants were consuming fewer food products with high PFUnDA contamination. Alternatively, these harder to explain findings could suggest that these regression analyses evaluating multiple food and drink categories may be more suited for exploratory analyses, which are hypothesis generating rather than providing definitive evidence of specific PFAS sources.

Even so, some of our main findings are consistent with the FDA findings from both regional and national Total Diet Sample studies, which have found evidence of PFAS contamination in fish (i.e., tilapia, cod, salmon, catfish, canned tuna), shellfish (i.e., shrimp), and meat (i.e., ground turkey, ground beef)^7^. While reports from these FDA studies conclude that there is no indication that the PFAS at levels identified in foods present a human health concern^20^, our study tested human samples and suggests that dietary sources of PFAS may be more problematic than previously thought. In addition to testing the food supply, future studies of human exposure to PFAS can help determine the key sources of these harmful chemical hazards. Reviewing the broader literature, there have only been a few studies assessing potential dietary sources of high PFAS exposure using food frequency questionnaires^21–23^. We believe that our study substantially adds to the literature by focusing on rural Americans, who may have distinctly different dietary patterns compared to urban Americans. The mean serum PFAS levels observed in our study population are generally comparable to those reported in National Health and Nutrition Examination Survey (NHANES)^24^. For example, our mean PFOS concentration (5.5 ng/mL) and mean PFOA concentration (1.5 ng/mL) are similar to geometric mean values reported in NHANES 2017–2018 (PFOS: 4.25 ng/mL; PFOA: 1.42 ng/mL). PFHxS (1.1 ng/mL) and PFNA (0.5 ng/mL) in our population were also comparable to NHANES estimates (PFHxS: 1.06 ng/mL; PFNA: 0.48 ng/mL).

This study has limitations. It was performed in Sullivan County, New York, and its findings may not be generalizable to other rural regions of the county. In addition, the years of water testing may not have aligned well with the duration of residence in the county among study participants. While water testing for PFAS contamination was available for those sources tested in our study, a more systematic analysis of water sources in the rural county might have yielded different results. Testing might have been more frequently performed in areas where there was existing concern and might have missed areas with PFAS water contamination that was not known. While PFAS water contamination was a concern, this study was not directly able to assess the drinking water consumed by individuals in the study. Therefore, the ecological analysis done on drinking water of this study may not be indicative of water borne exposures to PFAS among our study participants. Similarly, the sample of study participants included in the analysis might not have been representative of the geographic areas studied. Overall, our study might be limited by small samples sizes, and more comprehensive testing of human samples might be required to yield more generalizable results. Furthermore, the dietary data in the study is subject to the limitations of self-reported data. It could be that some meaningful sources of dietary exposure to PFAS were not included as questions on the FFQ, and the dietary associations identified may be confounded by other factors. Our study may also be limited using FFQs, which may not be the most accurate approach to quantifying dietary exposure sources. It may be that total diet studies or duplicate diet approaches may be even better at determining potential exposures sources of PFAS and other chemical hazards. Additionally, our FFQ did not capture the source of vegetables consumed (e.g., home-grown, store-bought, canned, or fresh). This is a limitation, as contaminant levels may vary considerably by source. Future studies should incorporate questions on food source to better characterize exposure pathways.

In conclusion, our study highlights the possibility that dietary sources of PFAS exposure may have a more significant impact on human health than previously thought, especially in areas where high water contamination might not be present. We also identify potential dietary sources of PFAS exposure that should be further explored, especially for rural communities that might have a different risk profile for PFAS exposure than residents in urban areas of the United States.

## Methods

### Study setting and participants

Residents of Sullivan County, New York, a region known to have poor health outcomes based on the county health rankings, were invited to participate in a multi-phase NIH-funded research study^25^. Sullivan County, New York has a population of 80,540, and the median household income is $72,382 (2020–2024) with a 18.7% poverty rate. A majority (82%) of the residents are white^26^. In the first phase, residents were contacted by mail to participate in a brief health survey, followed by a second phase in which respondents provided more detailed socioeconomic, health, and dietary data through more detailed surveys. In the third phase, participants were invited to provide blood and urine samples to measure potential dietary chemical exposures. Participants were recruited from 2022 to 2023 and provided Informed consent. Results of the chemical analyses were reported back to study participants with a report that included information about each chemical, their individual levels along with percentile compared to the study population, information about potential sources, and ways to avoid future exposure to these chemical hazards. This study was approved by the Institutional Review Board at the NYU School of Medicine under study protocol: s19-01920. Research was conducted in accordance with the Declaration of Helsinki. All study participants provided informed written consent.

### Primary outcome

Our primary outcome was the level of human exposure to PFAS, which was determined by chemical analyses using liquid chromatography coupled to mass spectrometry (LC-MS/MS). After blood was drawn and centrifuged, serum was aliquoted into freezer-safe polyethylene tubes, immediately placed on dry ice, and transported to -80 degrees Celsius storage until they were analyzed. The panel of PFAS chemicals tested included PFOSA, PFBS, PFHxS, PFOS-2, PFHxA, PFHpA, PFOA, PFNA, PFDA, PFUnDA, PFDoDA, N-MeFOSAA, and N-EtFOSAA. For samples with a value lower than the limit of detection (LOD), which ranged from 0.010 to 0.028, we used a value equal to the LOD divided by the square root of 2. We assessed quality control by testing reagent blanks, reagent spikes, and standard reference serums. These analyses were performed at the Human and Environmental Exposure Assessment Lab (HEAL), which is a specialized research lab for measuring environmental chemical exposures.

The extraction and analysis protocol were adapted from a previous study^27^. Quantification was performed using the isotope dilution method; all serum samples, reference materials, and procedural blanks were fortified with 50 µL of an isotope-labeled internal standard mixture (5.00 ng of each compound per 250 µL serum), and each target PFAS was quantified against its corresponding isotopically labeled analog. Method accuracy was assessed using NIST fortified human serum Standard Reference Materials (SRM 1957 and SRM 1958); measured concentrations for analytes with certified or reference values were within 20% of the assigned values. For analytes lacking certified values in these SRMs, method recovery was determined through fortified deionized-water samples extracted and analyzed alongside the serum samples, with recoveries falling within 20% of nominal spike concentrations. Procedural blanks, consisting of deionized water fortified with the isotope-labeled internal standard mixture and carried through the entire sample preparation procedure, were analyzed with each batch to assess potential contamination and analyte carryover. System blanks (neat methanol injections) were run at regular intervals to monitor instrumental background. To minimize potential contamination from the solid-phase extraction step, hybrid SPE-phospholipid cartridges were preconditioned with 1 mL of methanol containing 1% ammonium formate (w/v), and the wash effluent was removed by centrifugation prior to sample loading. All solvents, chemicals, and reagents used were of analytical grade. Analyses were performed on an ultra-high-performance liquid chromatography system coupled to an electrospray triple-quadrupole tandem mass spectrometer (UHPLC-ESI-MS/MS; ExionLC—Triple Quad 5500 + ; AB SCIEX, Framingham, MA, USA), with chromatographic separation achieved on an Acquity UPLC BEH C18 column (1.7 µm, 50 × 2.1 mm; Waters, Milford, MA, USA). The system was operated in negative-ion electrospray mode using compound-specific multiple reaction monitoring (MRM) transitions for both native and labeled analytes to ensure selective and sensitive detection.

### State water testing data

We requested all water PFAS data available from the New York State Department of Health for sources tested in Sullivan County, New York. The data included testing results from over 100 sites (i.e., apartments, condominiums, markets, day care centers, hotels, agricultural sites, medical facilities, mobile home parks, municipal water facilities, residential areas with well water, restaurants, and schools), between 2020 to 2024. We took the average levels of PFOA and PFOS across sites for each geographic area (either town or village) within the rural county. Data provided by the state health department were only available for these two types of PFAS chemicals in the water testing data. On average, the LOD for these water tests was approximately 2 ng/L.

### Health survey and dietary data

Each study participant answered a detailed health survey, which included demographic and socioeconomic information, such as age, sex, race/ethnicity, household type, estimated household income, highest level of education, disability status, the need for income support (e.g., social security income), the need for food support (e.g., food stamps), self-reported food insecurity, self-reported housing insecurity, difficulty meeting essential expenses, and difficulty meeting medical expenses. In addition, study participants provided in depth dietary information completing the Block food frequency questionnaire (FFQ), which included the frequency of intake for 129 specific categories of food and beverages (e.g., breakfast sandwiches, pizza, oysters, cakes, milk, energy drinks, soda). Participants also reported their distance to the nearest food store, frequency of canned food intake, and frequency of plastic wrapped food intake.

### Statistical analysis

Descriptive characteristics for study participants were summarized for the overall study population, along with upper, middle, and lower tertiles of total PFAS exposure, which summed the absolute levels of each PFAS chemical in nanograms per milliliter (ng/mL). These characteristics included total PFAS level, age, sex, race/ethnicity, household composition, household income, and highest level of education obtained. Differences between PFAS level tertiles along these factors were tested either by ANOVA for continuous variables or Fisher’s exact tests for categorical variables.

To map the geographic distribution of PFOS and PFOA contamination in water sources, we took the most recent levels for each site tested and averaged them with towns and villages within Sullivan County. For PFOS and PFOA exposure data from human samples, we took the average levels within the same towns and villages. We drew maps depicting five quintiles of exposure levels and performed Pearson correlation analyses to compare the geographic distribution of water contamination against human exposure data for each PFAS chemical.

To identify demographic, socioeconomic, and dietary factors associated with high PFAS levels, we performed a multivariable regression analysis using LASSO (least absolute shrinkage and selection operator) given the number of variables included in the analyses. Predictor variables included demographic and socioeconomic variables, along with more detailed socioeconomic data on income or food support, food or housing insecurity, difficulty meeting essential or medical expenses. Frequency data on dietary intake of 129 specific food and drink categories were included from the FFQ along with self-reported distance to the nearest food store, and frequency of canned food or plastic wrapped food intake. To normalize the PFAS exposure data, we used a log transformation for the primary outcome and also for each of the PFAS chemicals identified in the majority of study participants. To identify the most salient features, we reported predictors with high LASSO coefficients, choosing those that had a magnitude of at least 0.05.

Statistical analyses were performed using Stata 18.5 (Statacorp; College Station, TX, 2023). Geographic analysis and mapping were performed using ArcGIS Pro 2.8.3 (ESRI; Redlands, CA, 2021).

## Data Availability

The datasets generated and/or analyzed are available from the corresponding author on reasonable request. Interested parties can contact the study’s corresponding author at: david.lee@nyulangone.org.
